# Dynamic Monitoring of Systemic Biomarkers with Gastric Sensors

**DOI:** 10.1002/advs.202102861

**Published:** 2021-10-28

**Authors:** Christoph Steiger, Nhi V. Phan, Hen‐Wei Huang, Haoying Sun, Jacqueline N. Chu, Daniel Reker, Declan Gwynne, Joy Collins, Siddartha Tamang, Rebecca McManus, Aaron Lopes, Alison Hayward, Rebecca M. Baron, Edy Y. Kim, Giovanni Traverso

**Affiliations:** ^1^ Koch Institute for Integrative Cancer Research Massachusetts Institute of Technology Cambridge MA 02139 USA; ^2^ Division of Gastroenterology Brigham and Women's Hospital Harvard Medical School Boston MA 02115 USA; ^3^ Division of Gastroenterology Massachusetts General Hospital Harvard Medical School Boston MA 02115 USA; ^4^ Division of Comparative Medicine Massachusetts Institute of Technology Cambridge MA 02139 USA; ^5^ Division of Pulmonary and Critical Care Medicine Department of Medicine Brigham and Women's Hospital, Harvard Medical School Boston MA 02115 USA; ^6^ Department of Mechanical Engineering Massachusetts Institute of Technology Cambridge MA 02139 USA

**Keywords:** biomarker monitoring, continuous monitoring, gastric sensors, nasogastric tubes

## Abstract

Continuous monitoring in the intensive care setting has transformed the capacity to rapidly respond with interventions for patients in extremis. Noninvasive monitoring has generally been limited to transdermal or intravascular systems coupled to transducers including oxygen saturation or pressure. Here it is hypothesized that gastric fluid (GF) and gases, accessible through nasogastric (NG) tubes, commonly found in intensive care settings, can provide continuous access to a broad range of biomarkers. A broad characterization of biomarkers in swine GF coupled to time‐matched serum is conducted . The relationship and kinetics of GF‐derived analyte level dynamics is established by correlating these to serum levels in an acute renal failure and an inducible stress model performed in swine. The ability to monitor ketone levels and an inhaled anaesthetic agent (isoflurane) in vivo is demonstrated with novel NG‐compatible sensor systems in swine. Gastric access remains a main stay in the care of the critically ill patient, and here the potential is established to harness this establishes route for analyte evaluation for clinical management.

## Graphical abstract

Nasogastric tube placement in intensive care settings is a mainstay in the management of critical conditions. The authors demonstrate that systemic biomarkers are present in gastric fluid and that nasogastric tubes can be leveraged to monitor systemic disturbances through evaluation of these biomarkers. Real‐time biomarker monitoring can help improve responsiveness of clinical teams and improve critical care.

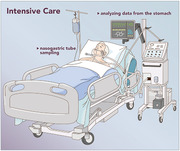

## 1 Introduction

Intensive care monitoring has increased the responsiveness of clinical care teams and improved the standard of care for patients in extremis.^[^ ^1^ ^]^ Major milestones that lead to fast responding professionals in modern intensive care units (ICU) include cardiac catheterization and photoplethysmography, which gained wide clinical implementation in the 1970s and 1980s.^[^ ^1^ ^]^ Photoplethysmography measures a variety of now commonly assessed parameters, including oxygen saturation. Today various systems are available to continuously monitor an array of vital signs including oxygenation, cardiac waveforms, hemodynamics (for example, blood pressure, heart rate), and temperature.^[^ ^1^ ^]^ Readouts can be automatically assessed through transdermal or intravascular access, and subsequently monitored in real time. Additionally, patients in the ICU generally require routine and frequent blood tests to monitor and detect complications, such as kidney failure or sepsis (delayed diagnosis of sepsis increases the mortality rate by up to 7.6% per hour^[^ ^2^ ^]^). Due to financial and clinical constraints the current sampling time resolution, however, can be too limited to enable an accurate response to the rapidly evolving clinical situation.^[^ ^3^ ^]^ Specifically, clinical chemistry analysis requires significant capital infrastructure that can often take a number of hours to report results back to the clinician. Additionally, repeated phlebotomy and intravenous access expose patients to a number of potential complications: iatrogenic anemia,^[^ ^4^ ^]^ bleeding, vascular injury (such as to the radial artery for arterial lines or to the carotid artery for central lines), digital ischemia, and infections.^[^ ^5^ ^]^ The latter is of particular concern: about 41 000 central line‐associated bloodstream infections (CLABSI) occur a year in the United States, 18 000 of which occur in ICUs.^[^ ^6^ ^]^ Each CLABSI costs up to $56 000 and contributes an additional 3 weeks to hospital length of stay and has a mortality rate of 14–40%. Breath diagnosis can help monitor relevant biomarkers, but it is limited to gaseous markers.^[^ ^16^ ^]^ Thus, there is a critical need to develop noninvasive alternatives to monitor serum derived biomarkers in real time.

**Figure 1:**
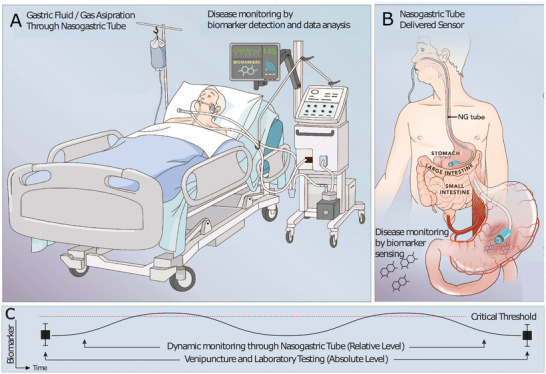
Schematic diagram demonstrating the concept to monitor systemic biomarkers with nasogastric (NG) compatible sensors. Gastric fluid (GF)/gas contains systemic biomarkers, and these can be monitored through A) aspiration and continuous analysis with ex vivo systems or B) intragastric sensor placements via NG tubes. The sensor resides in the stomach and is immersed in GF which contains systemic biomarkers. C) Demonstrates a use case in which a biomarker is monitored throughout the time period in between classical clinical laboratory testing to increase responsiveness of clinical care teams.

We hypothesized that gastric fluid (GF), accessible through a nasogastric (NG) tube, could be expanded to monitor markers associated with disease in critical care settings (**Figure** **1**). NG tubes facilitate access to GF and gastric gas and are already widely used in intensive care settings for enteral feeding and bowel decompression.^[^ ^7^ ^]^ Glands within the stomach wall produce ≈1.5 L GF per day.^[^ ^8^ ^]^ This fluid contains a cocktail of digestive compounds, such as pepsin and hydrochloric acid. It also contains many analytes present in serum ^[^ ^9^ ^]^ including urea,^[^ ^10^ ^]^ ammonia,^[^ ^11^ ^]^ or cortisol ^[^ ^12^ ^]^ as well as intravenously administered antibiotics like metronidazole^[^ ^13^ ^]^ and clarithromycin.^[^ ^14^ ^]^ To better understand the coincidence of markers in serum and GF we first analyzed a broad array of metabolic biomarkers in serum and time‐matched porcine GF. We then established the relationship and kinetics of analyte levels in an acute setting (acute renal failure via renal artery ligation) and inducible stress model (adrenocortotropic hormone injection) via endoscopically aspirated GF. Although biomarker correlation is limited, we observed a subset of analytes supporting the potential to monitor dynamic changes of systemic biomarkers and integrated a gas sensor into a commercially available NG tube to demonstrate in swine the ability to monitor alterations of an inhaled anaesthetic agent (isoflurane). We further expanded our proof of concept, to include a tethered sensor capable of continuous ketone (acetone) sensing and demonstrated in swine the ability to monitor ketone levels in real‐time. Ketone bodies such as acetoacetate, 3‐beta‐hydroxybutyrate, or acetone are formed physiologically for example during alternative gluconeogenesis from fat metabolism.^[^ ^15^ ^]^ Acetone is often used in the diagnosis and monitoring of ketoacidosis arising from conditions such as diabetes, alcohol intoxication.^[^ ^15^ ^]^ Early clinical research suggests that acetone can be monitored to facilitate early diagnosis of sepsis and guide nutritional support of critically ill patients.^[^ ^16^ ^]^

## 2 Results and Discussion

We hypothesized that GF and gases accessible through NG tubes could provide constant access to a broad range of biomarkers. This would enable monitoring dynamic biomarker profiles to improve response times of clinical care teams in particular as compared to turnaround of standard laboratory blood testing.

### 2.1 Time‐Matched Gastric and Serum Analyte Evaluation

**Figure 2:**
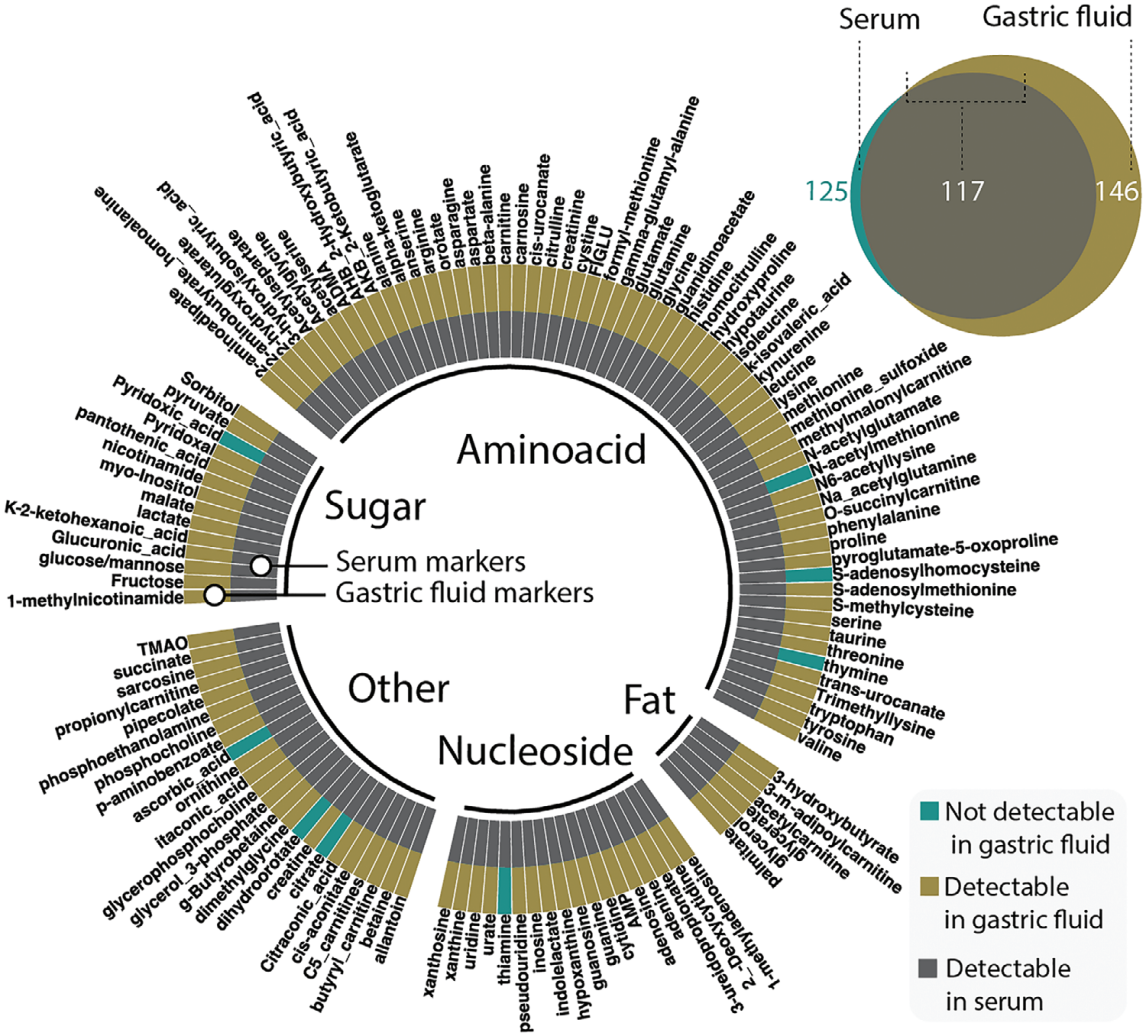
Coincidence of markers in serum and gastric fluid (GF). The plot shows a comparison of a set of analytes in serum‐ and GF. All but 8 out of 125 serum analytes (inner circle) are detectable in porcine GF (outer circle). For simplification the pie chart only shows detectable serum analytes. All markers are shown in the bubble chart (*n* = 5 for each group, 154 detectable markers in total).

To develop a NG tube compatible strategy, we sought to understand the qualitative and kinetic profile of biomarkers in GF and serum. Previous research on GF and gastric gases has largely focused on digestive properties and involvement in gastric diseases.^[^ ^8^, ^10^, ^11^, ^12^, ^13^, ^14^, ^17^ ^]^ The presence and kinetic profile of serum analytes, however, is not well understood. We conducted a time‐matched gastric and serum analyte evaluation and found that 94% of analyzed small molecular serum analytes can be found in GF (**Figure** **2**).

**Figure 3:**
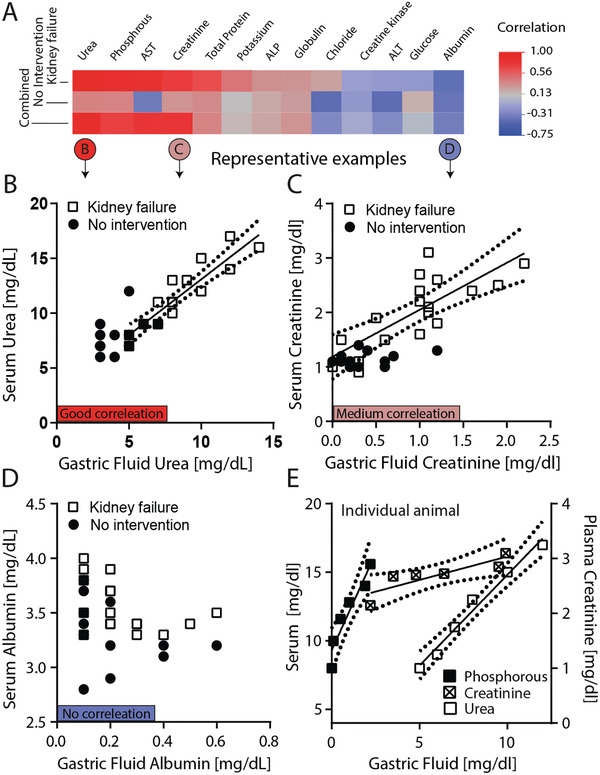
Correlation of biomarker levels in GF and serum withdrawn from nondiseased pigs (no intervention) and pigs that underwent arterial ligation (kidney failure). A) Heatmap showing the Pearson correlation for i) the kidney failure group (*n* = 3, sampled for 5 h) and animals without intervention (*n* = 15). Examples are shown for B) good correlation (urea, *r* > 0.9), C) medium correlation (creatinine, *r* > 0.7) and D) no correlation (albumin, *r* < 0.5). Correlations within A representative animal are shown in E) for phosphorus (left axis), urea (left axis), and creatinine (right axis). The dashed lines indicate the 95% confidence interval.

To explore biomarker distribution, we correlated time matched levels of serum and GF and found that basal biomarker levels did not correlate well (**Figure** **3**A, “no intervention”). However, following renal artery ligation, biomarkers that are clinically used to assess kidney function including urea and creatinine correlate well with serum levels (Figure 3). Some electrolytes such as chloride or potassium are involved in GF secretion^[^ ^18^ ^]^ which is in line with our observation that serum chloride and potassium levels change following arterial ligation, whereas corresponding GF levels remained constant (Figure S1 and Table S1, Supporting Information). However, we observed that serum and GF biomarkers in individual animals correlated well (Figure 3E).

### 2.2 Time Resolved Gastric‐ and Serum Analyte Evaluation

Following our observation that a broad range of biomarkers can be accessed through GF during disease states, we evaluated kinetic biomarker profiles in large animal disease models.

#### 2.2.1 Acute Kidney Failure Model

**Figure 4:**
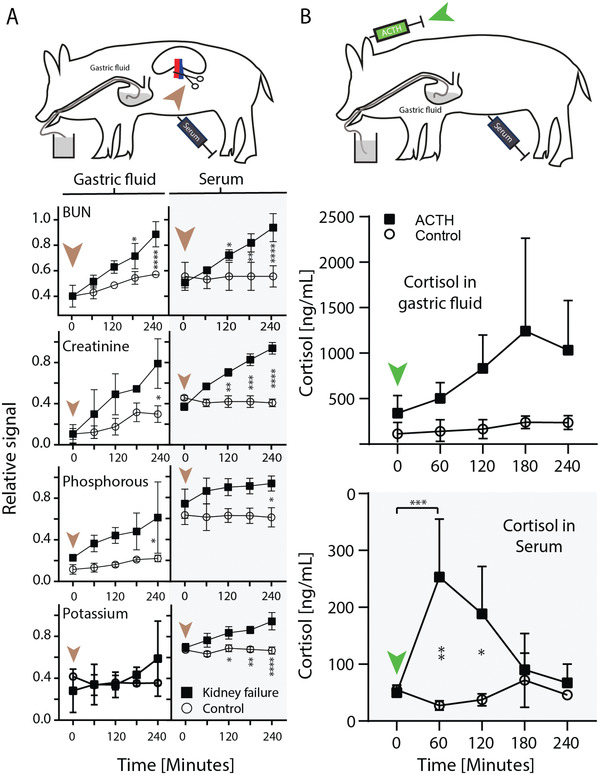
Biomarker dynamics in gastric fluid (GF) and serum. A) Porcine kidney failure model. The brown arrow indicates the induction of kidney failure by arterial ligation. The Asterix indicates the level of significance between the kidney failure group (squares) and the control group (circles). The control group did not undergo arterial clamping. B) Porcine inducible stress model. The green arrows indicate the injection of adrenocorticotropic hormone (ACTH). Results are depicted as mean of *n* = 3 ± SD (* *p* ≤ 0.05, ** *p* ≤ 0.01, *** *p* ≤ 0.001, **** *p* ≤ 0.0001.

Following arterial ligation clinically established renal markers including urea, creatinine, and phosphorous significantly increased in serum and GF over a time course of 4 h (**Figure** **4**A). Potassium levels increased significantly in serum, but not in GF (Figure 4A). Conversely, there was no significant difference among these markers at any timepoint in the control group (serum and GF), which comprised pigs that underwent hourly endoscopy for 4 h but no arterial ligation. The increase of biomarker levels was significant in the kidney failure group as compared to the control group for all renal markers, except potassium. In general, variability among biomarker levels was significantly higher in the GF group as compared to the serum group (pooled mean relative standard deviation: 20.2 ± 16.2% versus 6.8 ± 8.3%; *p* <0.0001, *n* = 78; Figure S1, Supporting Information).

#### 2.2.2 Inducible Stress Model

Adrenocorticotropic hormone (ACTH) physiologically triggers cortisol release from the adrenal cortex. Cortisol levels peaked within 1 and 3 h after injection of ACTH in serum and GF, respectively (Figure 4B). The levels returned to baseline in serum after 3 h, whereas they remained at 83 ± 44% of the peak value in GF after 4 h.

### 2.3 NG Tube Compatible Prototype for Biomarker Monitoring

The ease of administration, good intraindividual correlation and constant access to gastric content via NG tubes introduces the potential to continuously and noninvasively monitor dynamic profiles of biomarkers. We envisage two scenarios to translate these findings into a clinical tool: A) Aspiration of GF/gas for subsequent continuous ex vivo analysis or B) integration of sensors into NG tubes for in situ measurement. Continuous analytical methods with the capacity to gain high density data (such as selected‐ion flow‐tube mass spectrometry or ion mobility spectrometry) could be used for ex vivo analysis.^[^ ^19^ ^]^

Future, continuous intragastric sensor development could build on previous work in the area of ingestible and tethered sensors, which have specifically been modified for the gastrointestinal (GI) tract.^[^ ^20^ ^]^ For example, electrochemical liquid‐^[^ ^21^ ^]^ and gas phase^[^ ^22^ ^]^ sensing, spectrometric sensing,^[^ ^23^ ^]^ or bacterial–electronic sensing^[^ ^24^ ^]^ systems which have been previously described. Acetone is typically monitored in breath samples with semiconducting metal oxide sensors.

#### 2.3.1 Chemically Simulated Ketosis Model and Continuous Monitoring of Acetone

**Figure 5:**
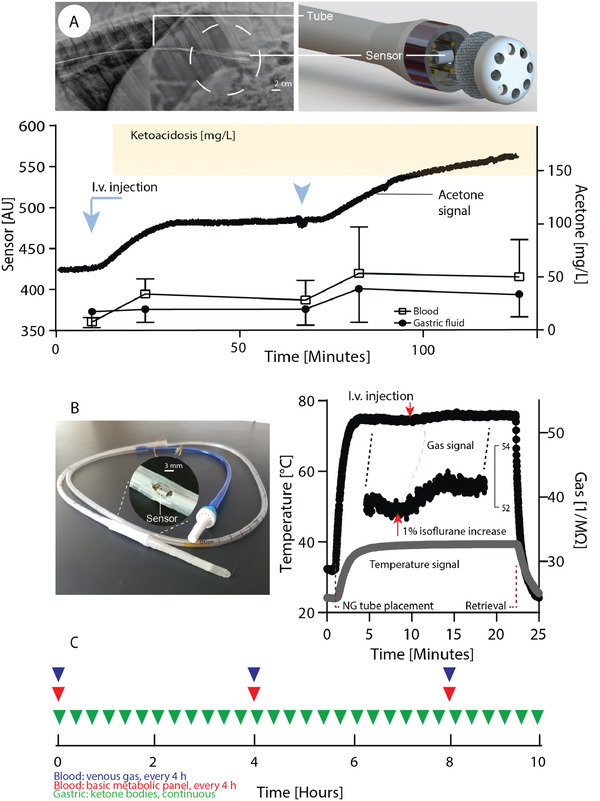
Figure Capacity of nasogastric (NG) tube compatible sensors to continuously monitor parameters for systemic health in swine. A) Chemically simulated ketoacidosis model. Acetone levels following intravenous injection in swine (indicated by the blue arrows) are monitored by an intragastric semiconductor sensor. The radiographic image shows the sensor after gastric placement. The sensor readout (left axis) over time is shown in comparison to acetone levels in blood and GF (right axis)—for comparison acetone levels commonly found in early ketoacidosis are shown (orange), accordingly.^[^ ^28^ ^]^ B) Commercially available NG tube comprising an integrated sensor with the capacity to simultaneously monitor multiple parameters (gas, temperature, pressure, humidity). Sensor readout following gastric placement in swine. The red arrow indicates increase of isoflurane flow from 2% to 3% for 5 min (used to maintain anesthesia). Results are depicted as mean of *n* = 4 for blood acetone ± SD, one reading is shown for the sensor readouts (see supplementary information for independent repeats as well as humidity / pressure data). C) Comparison of laboratory monitoring with phlebotomy versus gastric sensor over a typical course of diabetic ketoacidosis. Phlebotomy is typically done only every 4 h and provides information only about discrete time points, whereas gastric sensing could provide continuous information.^[^ ^25^ ^]^

Direct intragastric biomarker monitoring might address potential challenges associated with continuous sampling of GF and gases, such as tube blockage. As a proof of concept, we constructed a sensor system capable of residing in the stomach and continuously monitoring acetone, a ketone body and marker of ketoacidosis. The tethered sensor system was composed of a gas permeable polytetrafluoroethylene membrane and semiconductor gas sensor. The system was evaluated in vitro in porcine GF and demonstrated the capacity to detect acetone with a resolution of 3 µg L^−1^ (Figure S2 see Figure S3A for device, Supporting Information). We next evaluated the system in vivo demonstrating its capacity to sense intravenously‐administered acetone in GF with 60 min of administration (**Figure** **5**A). Consecutive injections of acetone resulted in a marked increase in sensor readouts which plateaued after ≈25 min (Figure 5A; and Figure S3B, Supporting Information). We performed gas chromatography mass spectrometry (GCMS) analysis to orthogonally assess acetone in blood and GF. Within 15 min blood acetone levels increased from 7.8 ± 4.5 to 34.3 ± 14.2 and 53.8 ± 43.0 mg L^−1^ for the first and second injection, respectively (Figure 5A). Within the same timeframe acetone levels in GF increased from 17.6 ± 14.9 to 19.7 ±12.5 and 39.3 ± 31.7 mg L^−1^, respectively (Figure 5A). We illustrate the potential clinical application of the sensor for management of diabetic ketoacidosis in Figure 5C. Compared to conventional management with serial phlebotomy every 2–4 h per guidelines from the American Diabetes Association, serial gastric sampling provide continuous insight into the patient's response to treatment, which may allow for more rapid titration of medication and faster resolution of ketosis.^[^ ^25^ ^]^

In addition to gas readouts we monitored intragastric temperature, pressure and humidity (integrated sensor, Figure S4, Supporting Information). Recording core body temperature is desirable given the low sensitivity of peripheral temperature to detect fever (64%).^[^ ^26^ ^]^ Continuous body temperature monitoring, in concert with blood tests, are central to early sepsis detection which is the cornerstone of modern sepsis therapy.^[^ ^27^ ^]^ While performing placement studies with the integrated sensor, we observed altered gas sensor readouts after we increased levels of isoflurane, applied per inhalation, by 1%. This serves as further support of the proof of concept to facilitate the clinical management through sensors residing in the stomach.

Limitation of these concepts, in particular with respect to its capacity to increase responsiveness of care teams, are demonstrated by the slow onset of gastric cortisol levels in the inducible stress model (*c*_max_: 60 and 180 min for serum and GF, Figure 4B). Previous data on the onset and elimination kinetics of blood derived compounds in GF is limited.^[^ ^29^ ^]^ Comparable data for saliva analysis, however, suggests that this process is highly variable and depends on various factors including chemical structure or dietary status (vide infra).^[^ ^30^ ^]^ Distribution kinetics require individual analysis for each analyte and species. And therefore, successful translation of our findings will require further characterization of a range of analytes in specific disease states and must also characterize potential confounders (e.g., depth of anesthesia) to inform optimal analytical setups, markers, or patterns.

Gastric tonometry, a technique that evaluates gastric carbon dioxide to assess tissue perfusion status, has been clinically used to help disease monitoring of critically ill patients^[^ ^31^ ^]^ and this might provide some evidence that quantitative analysis of gastric biomarkers can be performed despite the presence of disturbing factors, such as food.

Our data support the potential for gastric sensors to transform our capacity to monitor patients in acute settings. Multiple sensing concepts have been suggested to help diagnose and treat GI disease with ingestible sensors.^[^ ^20^ ^]^ For example, electrochemical liquid‐^[^ ^21^ ^]^ and gas phase^[^ ^22^ ^]^ sensing, spectrometric sensing,^[^ ^23^ ^]^ capacitive,^[^ ^32^ ^]^ or bacterial–electronic sensing ^[^ ^24^ ^]^ systems which have been previously described. Our data support our perspective that technical advancements in ingestible electronics can help improve biomarker monitoring concepts for systemic disease, accordingly. We demonstrate real time monitoring for systemic gaseous markers only (Figure 5). Our data on disease relevant systemic nongaseous markers (e.g., kidney disease relevant biomarkers, Figure 4) suggests that gastric sensing can open an avenue for noninvasive monitoring of nongaseous biomarkers which are currently not accessible through continuous monitoring approaches (e.g., breath analysis). Fully integrated sensor arrays for multiplexed biomarker monitoring in biofluids (e.g., sweat) that apply multiple sensing approaches simultaneously (e.g., electrochemical combined with enzymatic methods) have been described ^[^ ^33^ ^]^ and could be leveraged to monitor systemic biomarkers from GI fluids, accordingly (e.g., kidney disease relevant biomarkers).

Sepsis would be one use case given the distinct need for immediate therapy onset.^[^ ^2^ ^]^ In this study we used semiconductor sensors which can be configured in arrays composed of variable doped materials. In combination with temperature modulation such arrays can reach high selectivity and sensitivity.^[^ ^34^ ^]^ They can also be used to detect specific biomarker patterns: For example, this could be a pattern that is characteristic for the onset of sepsis and characterized by increasing acetone levels (accumulation of ketone bodies) in concert with increasing carbon dioxide levels (gastric tonometry, caused by hypoperfusion of splenic arteries). Clinical research is required to help understand characteristics of systemic biomarkers in GI fluids in humans to increase the clinical‐translational significance of this concept.

## 3 Conclusion

Gastric access remains a main stay in the care of the critically ill patient, and here we present the potential to harness this established route for analyte evaluation for clinical management. We envision alternative locations for tethered sensors (e.g., rectal sensors) as well as untethered ingestible gastric resident systems.^[^ ^35^ ^]^ The latter might further allow biomarker monitoring even outside of hospital settings.

## 4 Experimental Section

### Porcine In Vivo Studies

All procedures were conducted in accordance with the protocols approved by the Massachusetts Institute of Technology Committee on Animal Care. In vivo studies were performed in female Yorkshire pigs. The experiments were not blinded or randomized. The sample size was based on prior proof‐of‐concept studies on GI sensor systems.^[^ ^24^, ^36^ ^]^ Prior to any procedure, the animals were placed on a liquid diet (Ensure, Abbot Laboratories) for 24 h and then fasted overnight. Anesthesia was induced via intramuscular injections of 5.00 mg kg^−1^ Telazol (tiletamine/zolazepam, Decra Veterinary Products), 2.00 mg kg^−1^ Xylazine ( Bimeda), and 0.04 mg kg^−1^ atropine (Med Pharmex). The pigs were then intubated and anesthesia was maintained using inhaled isoflurane (1–3%, Patterson Laboratories).

GF samples were collected endoscopically (EG‐3470K gastroscope from Pentax, Tokyo) and immediately neutralized with sodium hydrogen carbonate (indicated by mColorpHast pH Test Strips, both from Sigma‐Aldrich). The neutralized GF was consecutively filtered through Acrodisc syringe filters (Pall laboratories; 5 and 0.2 µm, respectively) and then snap frozen with dry ice. Blood samples were stored in serum separator tubes (Vacutainer from BD) until they were centrifuged for 12 min at 2000xg. Serum and GF samples were stored at −80 °C.

### Biomarker Comparison in Serum and GF

GF was collected endoscopically along with blood samples in five pigs (4–8 months, weighing ≈40–70 kg). Minimum pig weight was determined in another study performed in the lab in which traces of blood in gastric fluid from pigs weighing ≈30 Kg after repeated gastroscopy were observed. Blood was not observed in gastric fluid derived from pigs weighing 40–70 kg. To address a potential bias by food intake, pigs were placed on liquid diet for 24 h and then fasted overnight (vide supra). Also, GF was profiled for constituents that are contained in gross amounts in the liquid diet using LCMS (citrate, thiamine, and ascorbic acid). Absence of these compounds indicates that the diet is not present in GF. LCMS analysis for all constituents in GF was performed as previously described with minor modifications.^[^ ^37^ ^]^ In brief, 10 µL filtered and neutralized GF samples or serum samples were combined with 90 µL of a solution containing 50% methanol, 30% acetonitrile, 20% water, and 17 isotope‐labeled amino acids (Cambridge Isotope Laboratories, Inc.). The mixture was vortexed for 5 min and centrifuged at 4 °C for 10 min at 21 130 rcf. The supernatant was collected and stored at −80 °C until LCMS analysis. For LCMS analysis, a QExactive benchtop orbitrap mass spectrometer and a Dionex UltiMate 3000 UPLC system (both from Thermo Fisher Scientific) were used. Analytes were separated on a ZIC‐pHILIC analytical column (150 mm × 2.1 *μ*m, 5 *μ*m particle size; Merck Millipore) as previously described.^[^ ^37^ ^]^ The mass spectrometer was operated in full scan, polarity‐switching mode (spray voltage: 3.0 kV; capillary temperature: 275 °C; HESI probe temperature: 350 °C; sheath gas flow rate: 40 units; auxiliary gas flow:15 units; sweep gas flow: 1 unit). Compound identification was performed with XCalibur QuanBrowser 2.2 (Thermo Fisher Scientific) based on a library previously established by the Whitehead Institute Metabolite Profiling core facility. A marker detectable was considered when the marker was detectable in at least 4 out of 5 animals.

### Acute Kidney Failure Model and Basal Biomarker Levels

Kidney failure was induced in three pigs. The control group comprised another three pigs that underwent the identical anesthetic and endoscopic procedure but no surgery. Pigs in both groups were 6–8 months old and weighting ≈70–80 kg. After draping and cleaning the abdomen with alcohol a midline incision extending from the xiphoid to the pubis was performed to open the abdominal cavity in the kidney failure group. Both kidneys were accessed by retracting the GI tact atraumatically. With blunt dissection, the renal artery and vein were clamped with two hemostatic forceps each. Successful induction of ischemia was controlled optically by immediate discoloration of the kidneys after ligation. Blood and GF samples were then collected over a course of 5 h. The pigs were sacrificed with 390 mg pentobarbital sodium, 50 mg phenytoin sodium at 120 mg kg^−1^ IV (Euthasol). To analyze basal biomarker levels GF and serum was withdrawn from 15 animals that were used in other studies in the lab (4–7 months, weighing ≈40–70 kg). Sampling was performed following induction of anesthesia (vide supra) and before any other procedure was started. Biomarkers in serum samples as well as neutralized and filtered GF samples were analyzed by a commercial entity (IDEXX, Westbrook) using a Beckman Coulter AU680. Spiking experiments with the analytes mixed with GF were performed to assure linearity.

### Inducible Stress Model

ACTH induced cortisol release was performed in three pigs as previously described, with modifications.^[^ ^12^ ^]^ In brief, 0.25 mg Cortrosyn (Amphastar Pharmaceuticals) was injected intravenously. Blood and GF samples were then collected over a course of 4 h (vide supra). Cortisol levels in GF and serum were analyzed by use of an ELISA kit from DRG international (Springfield Township).

### Ketone Sensor Prototyping and Sensing

A commercially available MQ‐3 tinoxide sensor was used from Zhengzhou Winsen that was attached to a Masterflex (OD: 10.5 mm ID: 7.3 mm) tube from Cole Parmer for gastric placement. The interconnection between the sensor and the tube was epoxy resin from Environmental Technology which was drop casted into a 3D printed mold. The sensor was sealed with a J050A025A Advantec Polytetrafluoroethylene membrane from Cole Parmer to protect the tinoxide coil from bodily fluids. To facilitate gastric placement the tip of the sensor was covered with an ethylene propylene diene monomer rubber cap from McMaster‐Carr which had 5 drilled holes of 2 mm wholes for gas exchange.

The sensor was first profiled in vitro in 25 mL porcine GF to which 0.1 mL of an aqueous acetone solution (1/200 m/m) was injected consecutively at 37 °C (see Figure S2 for setup, Supporting Information). For in vivo studies, a sterile acetone solution (4.7% m/m) was prepared in a laminar air flow hood under sterile conditions from acetone (HPLC Plus grade from Sigma‐Aldrich) and saline from B.Braun. The solution was filtered twice with a 0.2 µm Acrodisc syringe filters from pall laboratories. Filter integrity was tested with a manual bubble point test after filtration. Before performing any measurement, the sensor was allowed to run in for 30 min under ambient conditions and another 15–30 min following gastric placement in pigs (4–8 months, weighing ≈40–80 kg). 25 mL acetone solution was then then injected intravenously twice with an interval of 60 min. More acetone was not able to inject due to ethical considerations. In a second set of animals, blood and time‐matched GF samples after acetone injection with gas chromatography mass spectrometry (GCMS) were analyzed. Headspace solid phase microextraction (HS‐SPME) was used to sample acetone from blood and GF. The samples were subsequently injected into a Hewlett Packard 5890 Series II gas chromatograph with a 5972 mass selective detector for analysis. The device was operated with an HP‐Innowax column (30 m × 0.25 mm I.D., 0.50 µm) with an injection port and transfer‐line temperature of 280 °C. The samples were introduced into a split‐splitless injector with a column head pressure of 15.5 psi and a split ratio of 20:1. Blood and GF samples were sampled using the HS‐SPME technique through manual injection using a carboxen‐PDMS fiber. All samples were spiked to 100 ppm of 2‐propanol as the internal standard and heated to 30 °C for at least 30 min before sampling. The SPME fiber was exposed to the headspace of the samples for 3 min to reach adsorption equilibrium. The sample was then injected into the GCMS and allowed to desorb in the injection port for 1 min.

### Integrated Sensor Prototyping and Sensing

A BME680 sensor was integrated from Bosch Sensortec (size: 3.0 × 3.0 × 0.93 mm^3^) into a dual lumen stomach tube (NG tube) from Covidien and connected the sensor to a microcontroller (Atmega328p). The printed circuit board (PCB) was designed using Autodesk Eagle, a circuitry simulation and PCB design software. A Bantam Tools Othermill tabletop CNC machine was used to mill the board out of FR‐1 (thin copper layered over a nonconductive phenolic resin, for layout see Figure S3c, Supporting Information). The final board was assembled with Adam Technologies strip socket, Jonard Tools 34 AWG wire wrapping wire, and soldered using RoHS compliant Chipquik flux core solder wire. All but one apertures of the NG tubes where filled with epoxy resin. The sensor was then integrated into the open aperture and covered using an Aeos ePTFE tube (511+/−.039” ID). The link between ePTFE tube and NG tube was sealed with parafilm (Bemis). Before performing any measurement, the sensor was allowed to run in for 30 mins under ambient conditions. The NG tube was placed into the stomach through a gastric overtube placed under endoscopic guidance (for initiation of in vivo studies in swine see details above, pigs were 4–8 months, weighing ≈40–80 kg). Approximately 10 min after gastric placement isoflurane flow (see above) was increased from 2% to 3% for 5 min. During one measurement connection to the sensor was lost after 10 min presumable due to a loose contact. In vitro performance of the BME680 to detect isoflurane was performed by injecting isoflurane into an enclosed desiccator (volume 2.3 L, air in the desiccator was stirred at 150 rpm). In between measurements the lid was removed, and isoflurane allowed to evaporate.

### Statistical Analysis

Graphpad Prism 8.0 (GraphPad Software) was used for analysis of variance (ANOVA, with Tukey's multiple comparison) in the kidney failure and stress model (assuming normal distribution). Correlation analysis was done with Jump 14 (SAS Institute). Two‐group‐comparisons were performed with Student's *t*‐test (Jump 14, two sided, assuming normal distribution). All data are reported as mean ± standard deviation unless specified otherwise. *p* < 0.05 was considered statistically significant. All measurements were taken from distinct samples unless otherwise noted. All authors had access to the study data and reviewed and approved the final manuscript.

## Conflict of Interest

C.S. and G.T. are co‐inventors on multiple patents or patent applications describing ingestible electronics and auxiliary systems. G.T. reports receiving consulting fees from Novo Nordisk. C.S. is employed by Bayer Pharmaceuticals. Complete details of all relationships for profit and not for profit for G.T. can be found at the following link: https://www.dropbox.com/sh/szi7vnr4a2ajb56/AABs5N5i0q9AfT1IqIJAE‐T5a?dl=0.

## Author Contributions

C.S. and G.T. conceived and designed the research. C.S., N.P., and H.S. performed the biomarker quantification. C.S., N.P., H.S., J.C., S.T., A.H. performed the in vivo pig experiments. C.S., H.H., N.P., D.G., R.M. performed prototyping of novel devices. R.M.B., E.Y.K., and G.T. reviewed the manuscript and experimental design guidance. C.S. and D.R. performed data analysis. C.S. and G.T. wrote the manuscript with contributions from D.R., R.M.B., E.Y.K.

## Supporting information

Supporting Information

## Data Availability

Research data are not shared.
