# Supplementary material for: Dynamic Monitoring of Systemic Biomarkers with Gastric Sensors
Source: Adv Sci (Weinh). 2021 Oct 28;8(24):2102861. doi: 10.1002/advs.202102861 (PMC8693042; doi:10.1002/advs.202102861)
Supplement: Supplementary file 1 — Supporting Information [file ADVS-8-2102861-s001.pdf]

WILEY-VCH

© 2021 Wiley-VCH GmbH

ADVANCED SCIENCE
Open Access

Supporting Information

for *Adv. Sci.*, DOI: 10.1002/advs.202102861

Dynamic Monitoring of Systemic Biomarkers with Gastric Sensors

*Christoph Steiger[1,3], Nhi V. Phan[1], Hen-Wei Huang[1,3], Haoying Sun[1], Jacqueline N. Chu[1,6], Daniel Reker[1,3], Declan Gwynne[1,3], Joy Collins[1], Siddartha Tamang[1], Rebecca McManus[1], Aaron Lopes[1], Alison Hayward[1,4], Rebecca M. Baron[5], Edy Y. Kim[5], Giovanni Traverso[1,2,3]**

Supporting Information

Dynamic monitoring of systemic biomarkers with gastric sensors

Authors: *Christoph Steiger[1,3], Nhi V. Phan[1], Hen-Wei Huang[1,3], Haoying Sun[1], Jacqueline N. Chu[1,6], Daniel Reker[1,3], Declan Gwynne[1,3], Joy Collins[1], Siddartha Tamang[1], Rebecca McManus[1], Aaron Lopes[1], Alison Hayward[1,4], Rebecca M. Baron[5], Edy Y. Kim[5], Giovanni Traverso[1,2,3]**

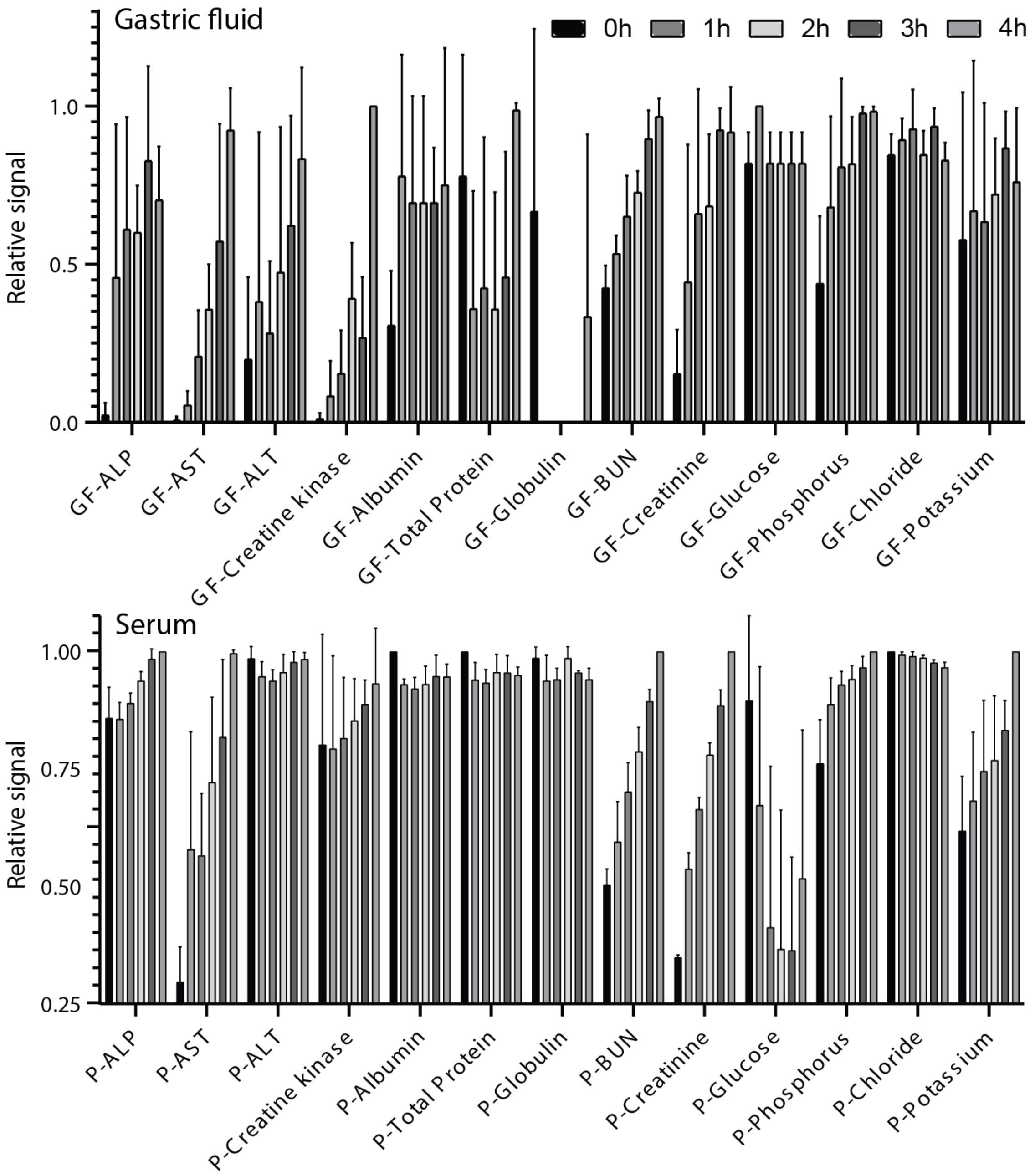

Figure S1: Porcine kidney failure model (all analytes). Kidney failure was induced by arterial ligation at timepoint 0. Sampling of gastric fluid and plasma was performed for 4 hours thereafter. Results are depicted as mean of n = 3 ± SD.

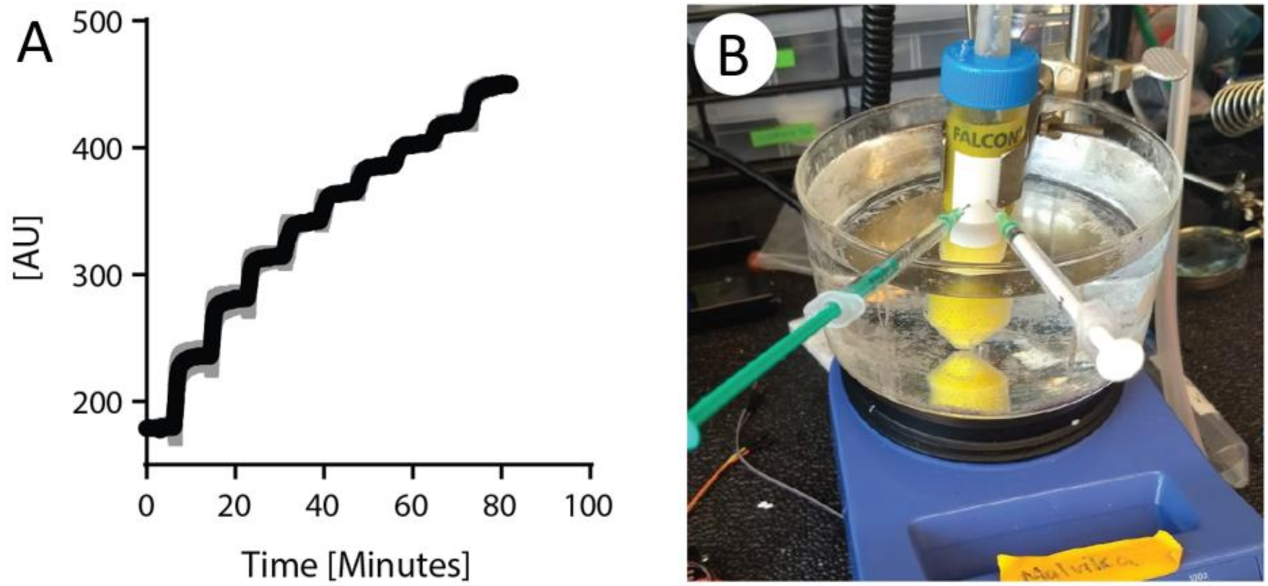

Figure S2: In-vitro performance of the Ketone sensor. A) sensor readouts after repeated injection of 78.5 ng acetone (1/200 (v/v) solution) into 25 mL porcine gastric fluid (n = 3 ± SD). Sensing was performed within a B) 50 mL falcon tube to which 25 mL porcine gastric fluid was added (37 °C). Results are depicted as mean of n = 3 ± SD.

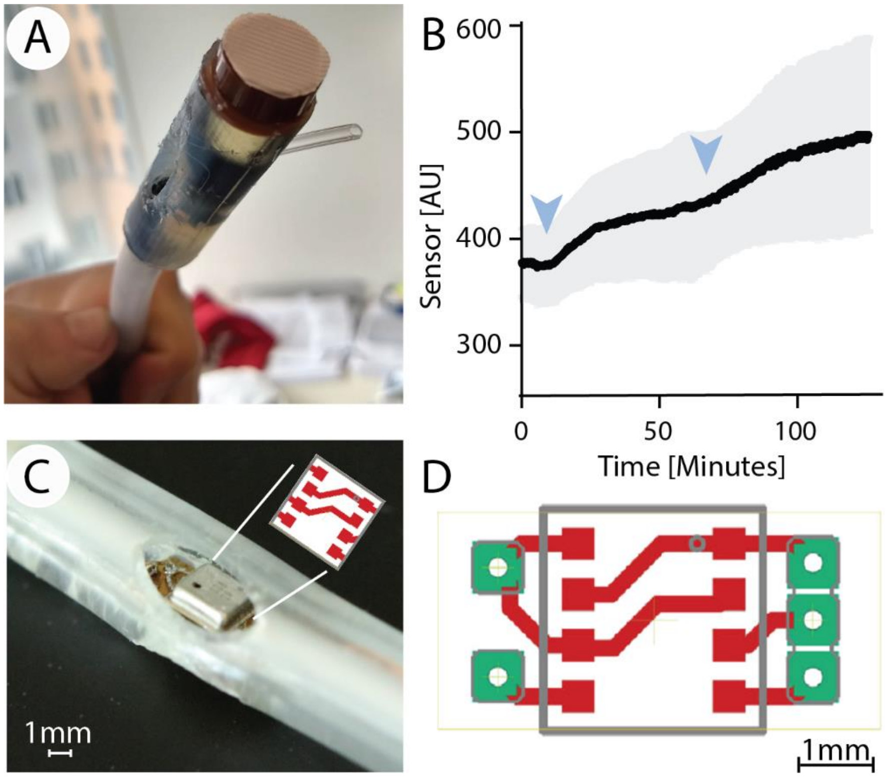

Figure S3: Nasogastric tube compatible sensor design A) Ketone sensor (without cap) and B) readouts following i.v. injection of acetone (indicated by the blue arrows) in a chemically induced ketoacidosis model (see Figure 4a for rest of dataset). C) Integrated sensor (within a commercially available NG tube). Sensor layout of the

integrated sensor (as indicated in C) is shown in detail in D). See Figure S4 for dataset. Results are depicted as mean of n = 3 ± SD.

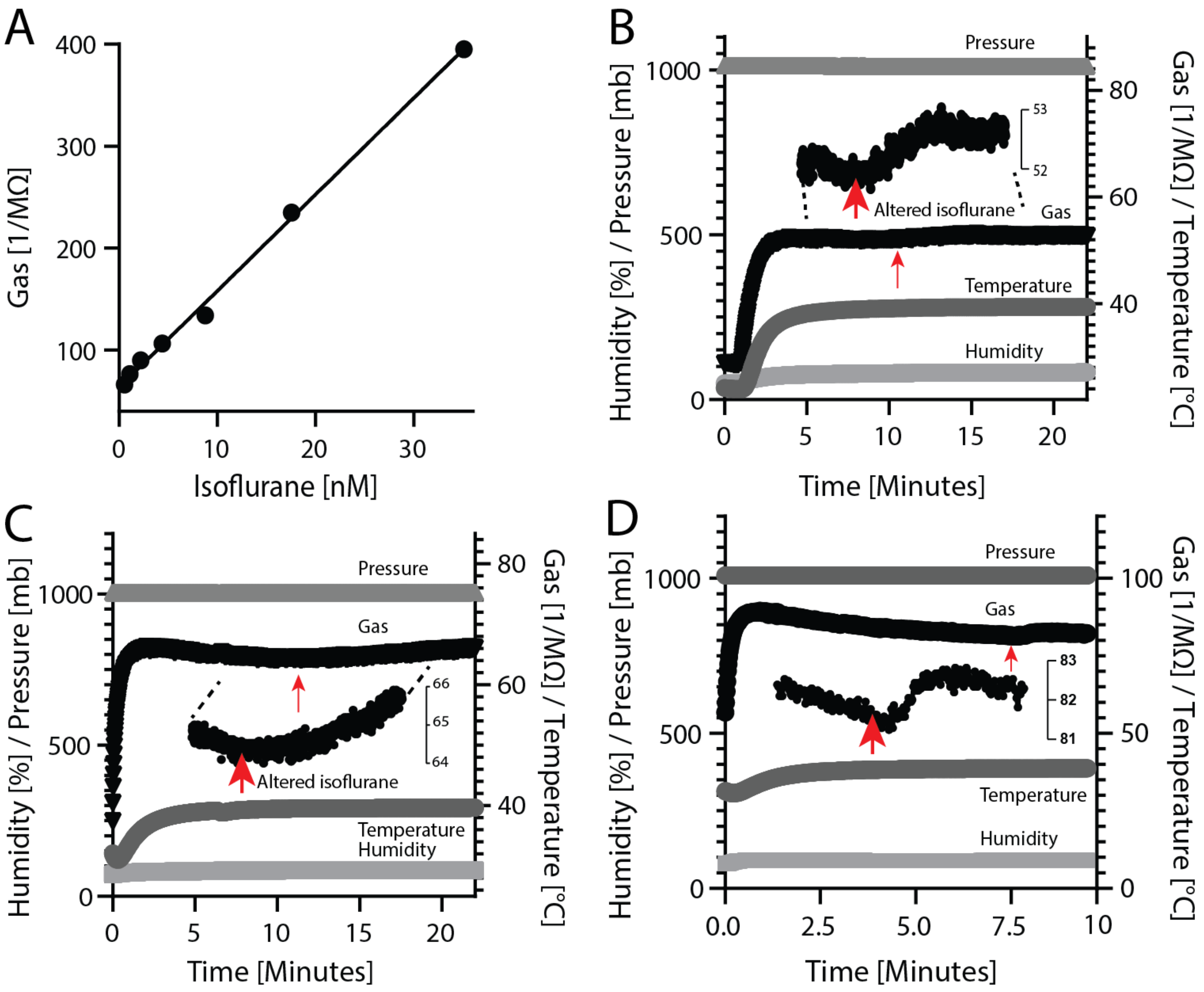

Figure S4: Nasogastric (NG) tube integrated sensor. A) Isoflurane calibration curve for the BME680 sensor integrated into NG tubes. Increasing quantities of isoflurane where directly pipette into a desiccator comprising the sensor (maximal readout for each injection is plotted against the isoflurane concentration, $r^2 = 0.997$). B) NG tube integrated sensor readouts (gas, pressure, humidity and temperature) following gastric placement in swine. The red arrow indicates increase of isoflurane flow from 2% to 3% (used Inhalational anaesthetic; C and D are replicates).

Table S1: Porcine kidney failure model (all analytes). Kidney failure was induced by arterial ligation at timepoint 0. Sampling of gastric fluid and plasma was performed for 4 hours thereafter. Results are depicted as mean of n = 3 ± SD.

**Gastric fluid**	**Average**					**Standard deviation**				
**Time [hour]**	**0**	**1**	**2**	**3**	**4**	**0**	**1**	**2**	**3**	**4**
ALP (U/L)	2.33	50.67	71.33	78.67	123.33	4.04	48.76	24.54	11.59	83.69
AST (U/L)	0.33	12.33	52.33	124.67	153.00	0.58	12.70	33.01	113.04	89.20
ALT (U/L)	1.00	1.67	2.67	3.67	6.67	0.00	0.58	2.89	2.08	4.51
Creatine kinase (U/L)	1.67	25.33	151.33	403.67	219.33	1.53	18.58	199.36	320.69	157.60
Albumin (g/dL)	0.10	0.27	0.23	0.23	0.30	0.00	0.12	0.06	0.06	0.20
Total Protein (g/dL)	30.43	22.57	28.53	22.43	21.53	52.37	39.00	49.33	38.77	37.12
Globulin (g/dL)	0.10	0.00	0.00	0.00	0.00	0.10	0.00	0.00	0.00	0.00
BUN (mg/dL)	5.00	6.33	7.67	8.67	10.67	0.00	0.58	0.58	1.15	1.15
Creatinine (mg/dL)	0.20	0.57	0.93	1.03	1.50	0.17	0.45	0.38	0.06	0.46
Glucose (mg/dL)	<10.00	12.33	<10.00	<10.00	<10.00	0.00	1.53	0.00	0.00	0.00
Phosphorus (mg/dL)	2.17	3.47	4.20	4.57	5.83	0.06	0.75	0.72	1.68	3.25
Chloride (mmol/L)	119.00	125.67	130.33	119.00	132.67	2.65	1.53	12.66	0.00	19.01
Potassium (mmol/L)	5.17	6.27	6.20	7.97	10.80	3.80	3.54	2.23	1.34	6.58

**Serum**	**Average**					**Standard deviation**				
**Time [hour]**	**0**	**1**	**2**	**3**	**4**	**0**	**1**	**2**	**3**	**4**
ALP (U/L)	110.33	110.00	114.33	120.33	126.33	13.65	10.58	9.07	6.66	9.50
AST (U/L)	19.33	35.67	37.00	51.00	59.00	5.03	4.73	9.85	24.33	29.72
ALT (U/L)	41.67	40.00	39.67	40.33	41.33	4.16	3.61	4.16	2.89	3.79
Creatine kinase (U/L)	547.33	544.33	566.33	596.33	629.33	80.65	51.47	13.43	50.50	125.60
Albumin (g/dL)	3.77	3.50	3.47	3.50	3.57	0.25	0.20	0.21	0.17	0.29
Total Protein (g/dL)	5.97	5.60	5.57	5.70	5.70	0.29	0.26	0.25	0.26	0.40
Globulin (g/dL)	2.20	2.10	2.10	2.20	2.13	0.17	0.30	0.20	0.20	0.21
BUN (mg/dL)	7.67	9.00	10.67	12.00	13.67	0.58	0.00	0.58	1.00	1.53
Creatinine (mg/dL)	1.00	1.53	1.90	2.23	2.53	0.10	0.06	0.10	0.15	0.15
Glucose (mg/dL)	85.33	91.67	67.33	59.33	51.67	58.65	113.78	97.58	85.45	67.04
Phosphorus (mg/dL)	11.37	13.23	13.80	13.97	14.33	2.14	1.89	1.25	1.14	1.10
Chloride (mmol/L)	97.33	96.67	96.33	96.00	95.00	1.15	1.53	2.08	1.00	1.73
Potassium (mmol/L)	4.23	4.67	5.10	5.27	5.77	0.25	0.40	0.36	0.21	0.49
